# P-glycoprotein-mediated chemoresistance is reversed by carbonic anhydrase XII inhibitors

**DOI:** 10.18632/oncotarget.13040

**Published:** 2016-11-03

**Authors:** Joanna Kopecka, Gregory M. Rankin, Iris C. Salaroglio, Sally-Ann Poulsen, Chiara Riganti

**Affiliations:** ^1^ Department of Oncology, University of Torino, 10126 Torino, Italy; ^2^ Eskitis Institute for Drug Discovery, Griffith University, Brisbane, Nathan, Queensland, 4111, Australia

**Keywords:** carbonic anhydrase XII, P-glycoprotein, doxorubicin, chemoresistance, intracellular pH

## Abstract

Carbonic anhydrase XII (CAXII) is a membrane enzyme that maintains pH homeostasis and sustains optimum P-glycoprotein (Pgp) efflux activity in cancer cells. Here, we investigated a panel of eight CAXII inhibitors (compounds 1–8), for their potential to reverse Pgp mediated tumor cell chemoresistance. Inhibitors (5 nM) were screened in human and murine cancer cells (colon, lung, breast, bone) with different expression levels of CAXII and Pgp. We identified three CAXII inhibitors (compounds 1, 2 and 4) that significantly (≥ 2 fold) increased the intracellular retention of the Pgp-substrate and chemotherapeutic doxorubicin, and restored its cytotoxic activity. The inhibitors lowered intracellular pH to indirectly impair Pgp activity. *Ca12*-knockout assays confirmed that the chemosensitizing property of the compounds was dependent on active CAXII. Furthermore, in a preclinical model of drug-resistant breast tumors compound 1 (1900 ng/kg) restored the efficacy of doxorubicin to the same extent as the direct Pgp inhibitor tariquidar. The expression of carbonic anhydrase IX had no effect on the intracellular doxorubicin accumulation. Our work provides strong evidence that CAXII inhibitors are effective chemosensitizer agents in CAXII-positive and Pgp-positive cancer cells. The use of CAXII inhibitors may represent a turning point in combinatorial chemotherapeutic schemes to treat multidrug-resistant tumors.

## INTRODUCTION

Carbonic anhydrases (CA, EC 4.2.1.1) are highly conserved and ubiquitous zinc metalloenzymes that reversibly hydrate carbon dioxide to bicarbonate and a proton: CO_2_ + H_2_O ⇆ HCO_3_^−^ + H^+^, controlling intracellular pH (pH_i_) [1]. CAIX and CAXII, two integral membrane proteins with the catalytic domain on the extracellular side, are overexpressed in many solid and hypoxic tumors [2–5]. Given the expression prevalence of CAXII in transformed cells, high levels of tissue-associated and circulating CAXII have been proposed as predictive markers of thyroid [6] and squamous lung [7] cancers, respectively. CAXII overexpression has also been associated with poor prognosis in human gliomas [8], oral squamous cancer [9] and esophageal squamous cell cancer [10]. In cancer cells CAXII contributes to extracellular acidification while also maintaining a normal intracellular pH (pH_i_). CAXII hydrates tumor cell generating CO_2_. As a consequence H^+^ ions are trapped extracellularly, lowering pH outside the cell (pH_o_), while HCO_3_^-^ is taken up by the cell to buffer the intracellular pH (pH_i_). [11, 12]. In healthy tissues CAXII is expressed mainly in the kidney and large intestine, with minimal expression in other tissues. The acid-base equilibrium regulated by CAXII is associated with Na^+^ and Cl^−^ transport that in turn drives passive water absorption in these organs [2, 13].

The inhibition of CAXII has been associated with impaired tumor growth, with a CAXII-inhibitory monoclonal antibody showing significant antitumor properties in mouse xenografts of breast cancer [14]. There is no corresponding study to correlate effects of small molecule CAXII inhibition with CAXII expression in tumors, however CAXII inhibitors have demonstrated antimetastatic activity in a breast cancer xenograft [15].

We recently demonstrated that CAXII is overexpressed in chemoresistant cancer cells expressing the drug efflux transporter P-glycoprotein (Pgp) [16]. Pgp recognizes multiple substrates, including a broad range of chemotherapeutics. Pgp expression in cancer cells contributes to multidrug resistance (MDR) [17, 18]. CAXII physically interacts with Pgp and it has been proposed that CAXII maintains the optimal pH for Pgp efflux activity, thus potentiating the contribution of Pgp to MDR [13].

Given the correlation of CAXII activity with tumor proliferation, invasion and chemoresistance, the interest in developing selective inhibitors of CAXII has increased in recent years, however *in vitro* and *in vivo* studies with small molecule CAXII inhibitors that directly link the mechanism of action to CAXII inhibition remain scarce and CAXII is much less studied than the other cancer-associated CA, CAIX.

The aim of this study is to evaluate CAXII inhibitors as selective chemosensitizers in MDR tumor models. Eight test inhibitors with variable CA inhibition profiles and variable physicochemical properties were selected to establish the potential of CAXII inhibitors to indirectly inhibit Pgp activity to resensitize MDR cells to doxorubicin. We show that CAXII inhibitors have very good chemosensitizing efficacy, and increase the effectiveness of the chemotherapeutic drug doxorubicin up to 4.4-fold. This correlated with high expression of both CAXII and Pgp *in vitro* and *in vivo*. Our results demonstrate that small molecule CAXII inhibitors may have future applications in clinical settings, for example, by allowing a reduction in the dosage of cytotoxic drugs or To restore the cytotoxic efficacy of existing drugs that have lost their effectiveness i.e. resensitizing of hard-to-treat tumors to chemotherapeutics.

## RESULTS

### Selective CAXII inhibitors increase doxorubicin accumulation and cytotoxicity in CAXII-positive and Pgp-positive cancer cells

**Figure 1:**
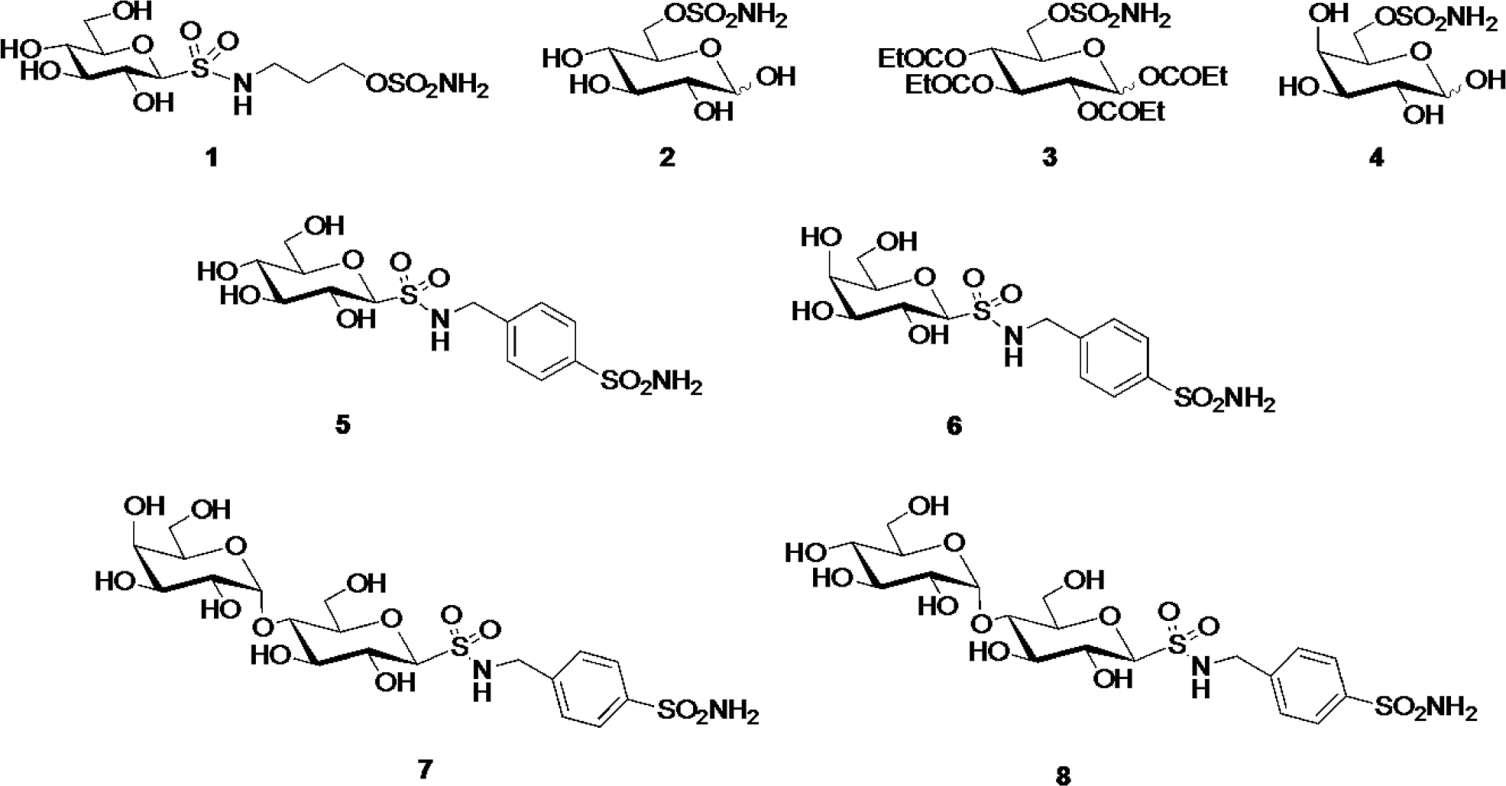
Chemical structure of CAXII inhibitors

The CA inhibition, CAXII selectivity and cLog *P value*s for inhibitors 1–8 (Figure 1) are given in Table 1.

**Table 1:** Inhibition data (*K*_*i*_) of human CA isozymes I, II, IX and XII, CAXII selectivity and cLog *P* values of compounds 1–8 and the established CA inhibitor acetazolamide (AZA)

Compd	cLog P^a^	*K_i_* (nM)^b^				CAXII Isozyme Selectivity^c^		
		CA I	CA II	CA IX	CA XII	CA I/XII	CAII/ XII	CA IX/XII
**1**	−2.65	9000	5.0	2.0	1.0	9000	5.0	2.0
**2**	−3.30	1180	82	8.6	7.3	161	11.2	1.2
**3**	+1.40	3360	105	77	96	35	1.1	0.8
**4**	−3.30	4500	93	62	7.6	592	12.2	8.2
**5**	−3.17	102	9.1	95	8.3	12.3	1.1	11.5
**6**	−3.17	87	7.8	99	8.7	10	0.9	11.4
**7**	−4.94	107	5.0	106	8.9	12.0	0.6	11.9
**8**	−4.94	101	4.8	98	8.2	12.3	0.6	12.0
**AZA**	−1.00	250	12	25	5.7	43.9	2.1	4.4

a Calculated using ChemDraw Ultra 12 or InstantJChem 3.0.4 from ChemAxon.

b Errors in the range of ± 5% of the reported value, from three determinations.

c Selectivity is determined by the ratio of *K*_i_s for CA isozyme relative to CAXII. Nonstandard abbreviations: cLog P (calculated Log P partician coefficient of the compound between octanol and water), *K*_i_ (inhibition constant).

**Figure 2:**
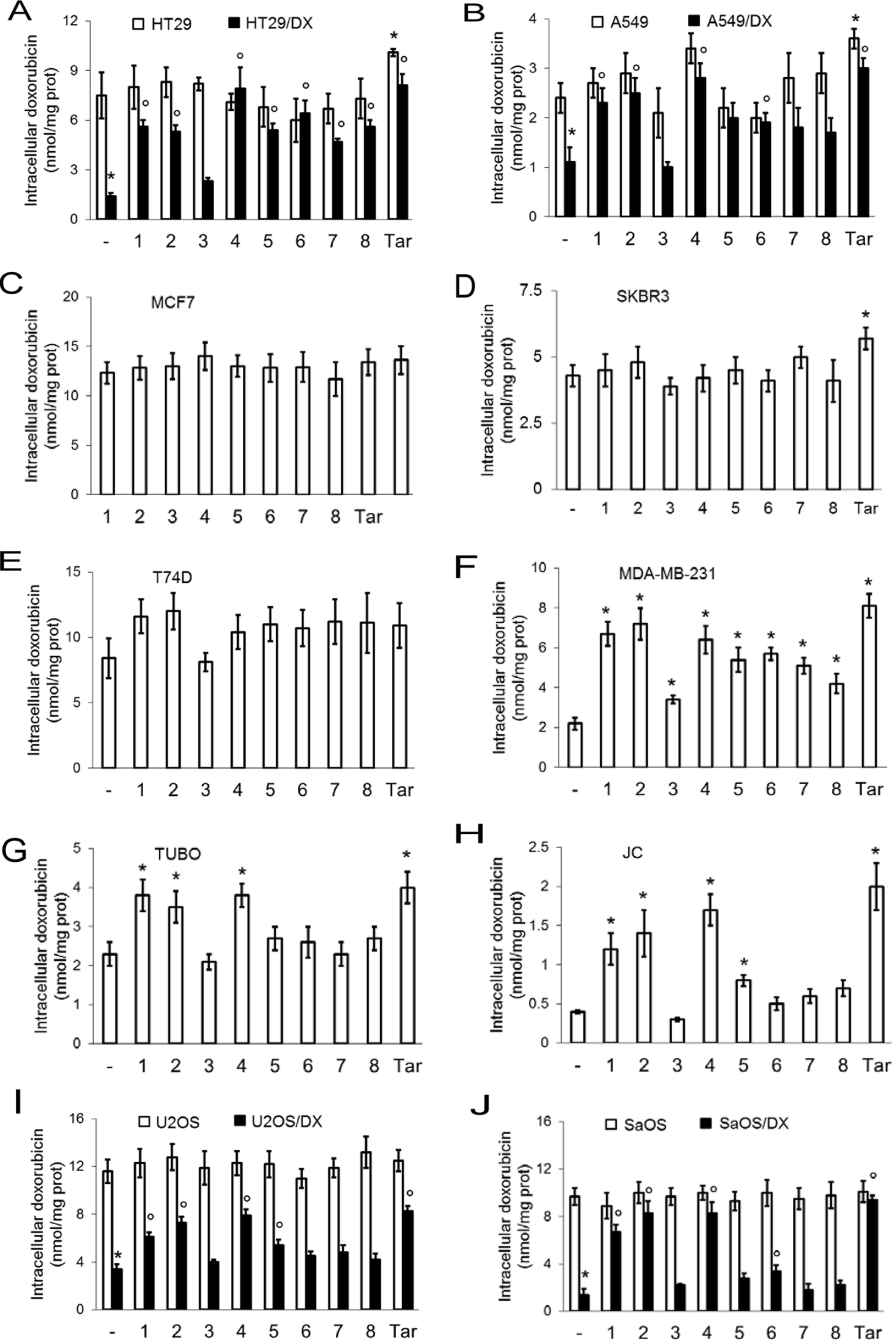
Effects of CAXII inhibitors on intracellular doxorubicin retention in drug-sensitive and drug-resistant cancer cells Human doxorubicin-sensitive colon cancer HT29 cells and their resistant counterpart HT29/DX cells (panel **A**), human doxorubicinsensitive lung cancer A549 cells and their resistant counterpart A549/DX cells (panel **B**), human doxorubicin-sensitive and resistant breast cancer MCF7 (panel **C**), SKBR3 (panel **D**), T74D (panel **E**) and MDA-MB-231 cells (panel **F**), murine doxorubicin-resistant TUBO (panel **G**) and JC cells (panel **H**), human doxorubicin-sensitive osteosarcoma U2OS cells and their resistant counterpart U2OS/DX cells (panel **I**), human doxorubicin-sensitive osteosarcoma Saos and their resistant counterpart SaOS/DX (panel **J**) were grown for 24 h in the presence of 5 μM doxorubicin, alone (–) or in the presence of 5 nM of compounds 1–8. Tariquidar (25 nM; Tar) was included as Pgp inhibitor. The intracellular drug content was measured fluorimetrically. Data are presented as means ± SD (*n* = 4). Versus doxorubicin alone (–): *p < 0.05; for cells treated with compounds 1–8 or tariquidar, doxorubicin-resistant cells versus the corresponding doxorubicin-sensitive cells: °p < 0.05.

On the basis of the *K*_i_ values for 1, 2 and 4–8 at CAXII (*K*_i_ < 10 nM), all the compounds were evaluated at 5 nM concentrations in the following *in vitro* assays. In these experimental conditions, compounds 1, 2 and 4 increased the intracellular accumulation of doxorubicin, a Pgp substrate, in cells with high expression of both CAXII and Pgp (Supplementary Figure S1), such as HT29/DX, A549/DX, MDA-MB-231, TUBO, JC, U2OS/DX and SaOS/DX cells (Figure 2A–2J). The compounds had no effect on cells with detectable levels of just one of these two proteins expressed (Supplementary Figure S1), such as HT29, A549, MCF7, SKBR3, T74D, U2OS and SaOS cells (Figure 2A–2J). The expression of CAIX did not influence the effects of the compounds on the intracellular doxorubicin accumulation in all cell lines tested. Compound 3 (CAXII *K*_i_ = 96 nM) has low activity as a CAXII inhibitor, however was included in the study as it is a prodrug of compound 2, an active CAXII inhibitor (CAXII *K*_i_ = 7.3 nM). Consequently compounds 2 and 3 provide a ‘drug/prodrug’ pair where the observed activity of the ‘drug’ may be predominantly attributed to 2. Compounds 1, 2 and 4 were the most consistently effective at increasing the intracellular accumulation of doxorubicin across all CAXII-positive and Pgp-positive cell lines tested, showing the same potency as the direct Pgp inhibitor, tariquidar (Figure 2A–2J). The compounds also increased the intracellular retention of the chemotherapeutics vinblastine (Supplementary Figure S2A) and paclitaxel (Supplementary Figure S2B), two Pgp substrates with different Pgp binding sites [19]. Compounds 1, 2 and 4 were selected for the follow-up assays, described next.

**Figure 3:**
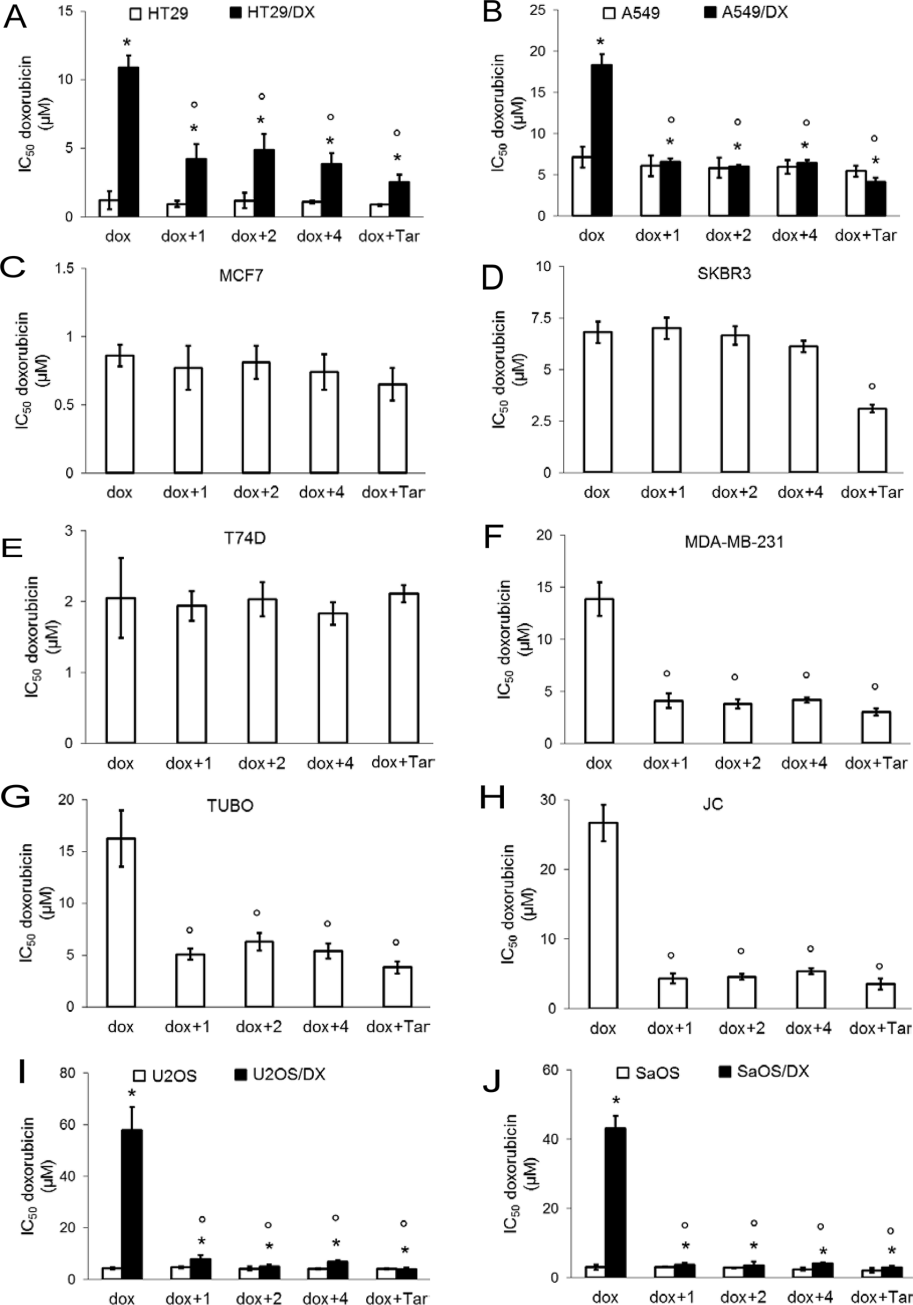
Effects of CAXII inhibitors on viability of drug-sensitive and drug-resistant cancer cells Human doxorubicin-sensitive colon cancer HT29 cells and their resistant counterpart HT29/DX cells (panel **A**), human doxorubicin-sensitive lung cancer A549 cells and their resistant counterpart A549/DX cells (panel **B**), human doxorubicin-sensitive and resistant breast cancer MCF7 (panel **C**), SKBR3 (panel **D**), T74D (panel **E**) and MDA-MB-231 cells (panel **F**), murine doxorubicin-resistant TUBO (panel **G**) and JC cells (panel **H**), human doxorubicin-sensitive osteosarcoma U2OS cells and their resistant counterpart U2OS/DX cells (panel **I**), human doxorubicin-sensitive osteosarcoma Saos and their resistant counterpart SaOS/DX (panel **J**) were incubated for 72 h with increasing concentrations (1 nM – 1 mM) of doxorubicin (dox), alone or in the presence of 5 nM of compound 1, 2 and 4, then stained in quadruplicate with neutral red. Tariquidar (25 nM; Tar) was included as a Pgp inhibitor. IC_50_ of tariquidar alone was > 10 μM; at 25 nM tariquidar reduced viability ≤ 8.23 ± 1.44 % in each cell line. Data are presented as mean IC_50_ ± SD (*n* = 4). HT29/DX, A549/DX, U2OS/DX, SaOS/DX versus HT29, A549, U2OS, SaOS cells: *p < 0.001; versus doxorubicin alone: °*p* < 0.005.

In accordance with the correlation of CAXII expression and cancer cell proliferation [14], compounds 1, 2 and 4 reduced the viability of CAXII-positive cell lines. The reduction in viability for individual compounds was: ≤ 31 ± 6% in HT29/DX cells, ≤ 28 ± 10% in A549 cells, ≤ 38 ± 7% in A549/DX cells, ≤ 33 ± 12% in T74D cells, ≤ 36 ± 11% in MDA-MB-231 cells, ≤ 28 ± 7% in TUBO cells, ≤ 30 ± 10% in JC cells, ≤ 27 ± 8% in U2OS/DX cells, ≤ 32 ± 7% in SaOS/DX cells (*p* < 0.05 for all cell lines; *n* = 4). In contrast, the compounds were devoid of any effects on viability in cells with low or undetectable levels of CAXII, including HT29, MCF7, SKBR3, U2OS, SaOS cell lines (not shown). As expected, doxorubicin reduced viability in cells with undetectable or low levels of Pgp, i.e. HT29, A549, MCF7, SKBR3, T74D, U2OS and SaOS cells; in these doxorubicin-sensitive cell lines the compounds did not exert additive effects on viability compared to doxorubicin treatment alone (not shown). In contrast, HT29/DX, A549/DX, MDA-MB-231, TUBO, JC, U2OS/DX, SaOS/DX cells, which are positive for both Pgp and CAXII (Supplementary Figure S1), were unresponsive to doxorubicin alone not shown. Compounds 1, 2 and 4 restored doxorubicin efficacy and further reduced cell viability. The differences in cell viability between cells treated with compounds alone and cells co-treated with compounds plus doxorubicin were: ≥ 38 ± 6% in HT29/DX cells, ≥ 22 ± 8% in A549 cells, ≥ 38 ± 7% in A549/DX cells, ≥ 18 ± 7% in T74D cells, ≥ 34 ± 10% in MDA-MB-231 cells, ≥ 22 ± 8% in TUBO cells, ≥ 29 ± 8% in JC cells, ≥ 27 ± 9% in U2OS/DX cells, ≥ 27 ± 7% in SaOS/DX cells, (*p* < 0.05; *n* = 4). These differences suggest that the decreased viability of cells co-treated with CAXII inhibitors and doxorubicin was due to the increased doxorubicin accumulation with added compound 1, 2 or 4 and/or to a synergistic effect of compound 1, 2 or 4 and doxorubicin, rather than to cytotoxicity exerted by the CAXII inhibitors themselves. Accordingly, the doxorubicin IC_50_ was significantly reduced by the co-treatment with the CAXII inhibitors in these cell lines. Co-treatment with compounds 1, 2 and 4 had the same efficacy as treatment with tariquidar (Figure 3A–3J) in resensitizing cells to doxorubicin (Table 2). Notably, in CAXII-negative MCF7 and SKBR3 cells that overexpress Pgp (Supplementary Figure S3A), the compounds did not increase the intracellular retention of doxorubicin (Supplementary Figure S3B). Lastly, compounds 1, 2 and 4 did not exert any cytotoxic effect (Supplementary Figure S4B) in not-transformed human epithelial colon CCD-Co-18 cells, epithelial lung BEAS-2B cells, epithelial breast MCF10A cells or fibroblasts that do not have detectable levels of CAXII (Supplementary Figure S4A). Collectively these results demonstrate that compounds 1, 2 and 4 are cytotoxic agents against CAXII-positive cancer cells and substantially reverse doxorubicin resistance in cancer cells expressing both CAXII and Pgp, but are without significant cytotoxicity in CAXII-negative and not-transformed cells.

**Table 2:** Resistance factor (Rf) of cell lines treated with doxorubicin alone or doxorubicin in the presence of compounds 1, 2 and 4, versus cells treated with doxorubicin *plus* tariquidar

Cell line	Rf dox	Rf dox + 1	Rf dox + 2	Rf dox + 4
HT29	1.39	1.07	1.35	1.23
HT29/DX	4.26	1.66	1.91	1.52
A549	1.31	1.21	1.08	1.09
A549/DX	4.45	1.59	1.46	1.57
MCF7	1.32	1.18	1.25	1.14
SKBR3	2.19	2.25	2.14	1.97
T74D	0.98	0.92	0.96	0.88
MDA-MB-231	4.55	1.34	1.25	1.37
TUBO	4.23	1.33	1.64	1.41
JC	7.61	1.23	1.29	1.54
U2OS	1.05	1.14	1.02	0.99
U2OS/DX	15.44	2.02	1.31	1.76
SaOS	1.43	1.47	1.36	1.18
SaOS/DX	14.97	1.32	1.26	1.42

Cells were incubated for 72 h with increasing concentrations (1 nM–1 mM) of doxorubicin (dox), alone or in the presence of 5 nM of compound 1, 2 and 4, then stained in quadruplicate with neutral red. Tariquidar (25 nM, for 24 h; Tar) was included as Pgp inhibitor (*n* = 4). Rf (resistance factor) was calculated as the ratio between mean IC_50_ in cells treated with compound 1, 2 and 4, and mean IC_50_ in cells treated with doxorubicin and tarquidar.

### Selective CAXII inhibitors lower Pgp ATPase activity and alter intracellular pH

**Figure 4:**
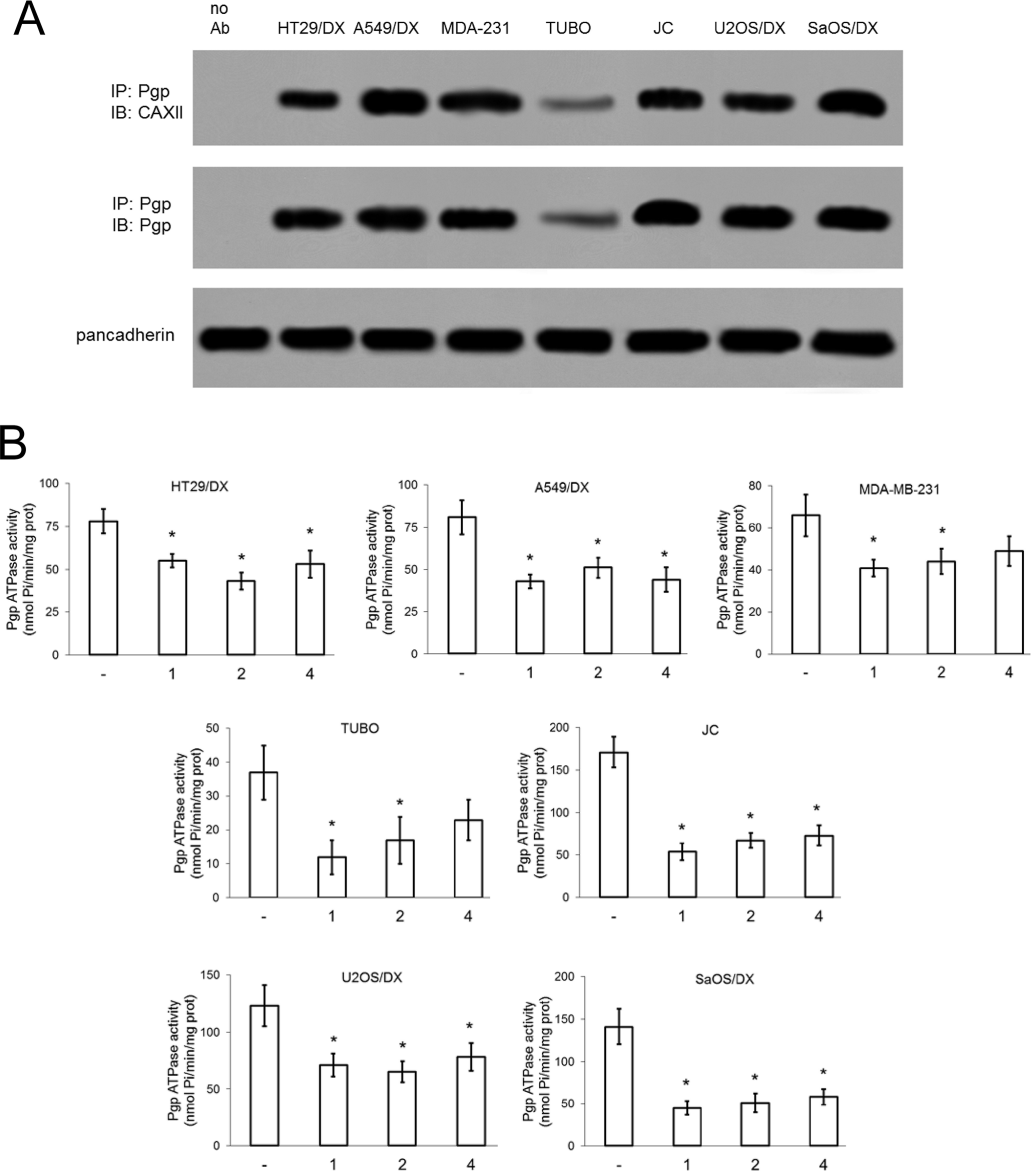
Effects of compound 1, 2 and 4 on Pgp activity (**A**) Biotinylated plasma membrane-derived extracts from HT29/DX, A549/DX, MDA-MB-231, TUBO, JC, U2OS/DX and SaOS/DX cells were immunoprecipitated (IP) with anti-Pgp antibody, then immunoblotted (IB) with anti-CAXII or anti-Pgp antibodies. Pancadherin level was used as control of equal protein loading. no Ab: A549/DX sample immunoprecipitated without antibody. The figure is representative of one out of three experiments with similar results. (**B**) Cells were grown for 24 h in fresh medium (–) or in medium containing 5 nM compounds 1, 2 and 4. The Pgp ATPase activity was measured spectrophotometrically on Pgp-rich vesicles extracted from membrane fractions. Data are presented as means ± SD (*n* = 3). For all cell lines, versus untreated cells (–): *p < 0.002.

CAXII and Pgp proteins co-immunoprecipitated (Figure 4A) in HT29/DX, A549/DX, MDA-MB-231, TUBO, JC, U2OS/DX and SaOS/DX cells, which express both CAXII and Pgp (Supplementary Figure S1), as well as in Pgp-enriched vesicles derived from the membranes of these cells (Supplementary Figure S5), indicating that these two enzymes were physically associated in the cell plasma membrane. Treatment of these doxorubicin-resistant cell lines with CAXII inhibitors 1, 2 and 4 (5 nM) lowered the pH_i_ relative to untreated cells: compound 1 (CAXII *K*_i_ = 1.0 nM) reduced pH_i_ ≥ 0.24 units, compound 2 (CAXII = *K*_i_ 7.3 nM) reduced pH_i_ ≥ 0.12 units, compound 4 (CAXII = *K*_i_ 7.6 nM) reduced pH_i_ ≥ 0.06 units (Supplementary Figure S6). Isolated Pgp has consistent basal activity across the pH range 6.2 to 7.6 (at 37°C) [16], this range encompasses the pH_i_ of the resistant cancer cell lines tested (Supplementary Figure S6). Notably, the reduction of pH_i_ exerted by compounds 1, 2 and 4 (Supplementary Figure S6) was paralleled by a modest, but significant reduction (≥ 1.47-fold) of Pgp ATPase activity (Figure 4B). The compounds also reduced the substrate-dependent activation of Pgp, as demonstrated by the ATPase assays performed in the presence of Pgp substrate doxorubicin (Supplementary Figure S7). In contrast, in the presence of the Pgp inhibitor tariquidar, which strongly decreased ATPase activity, there was no further reduction of ATPase activity by co-treatment of tariquidar with compounds 1, 2 and 4 (Supplementary Figure S7).

### Knockout of ca12 abrogates the effects of CAXII inhibitors on doxorubicin accumulation and cytotoxicity

**Figure 5:**
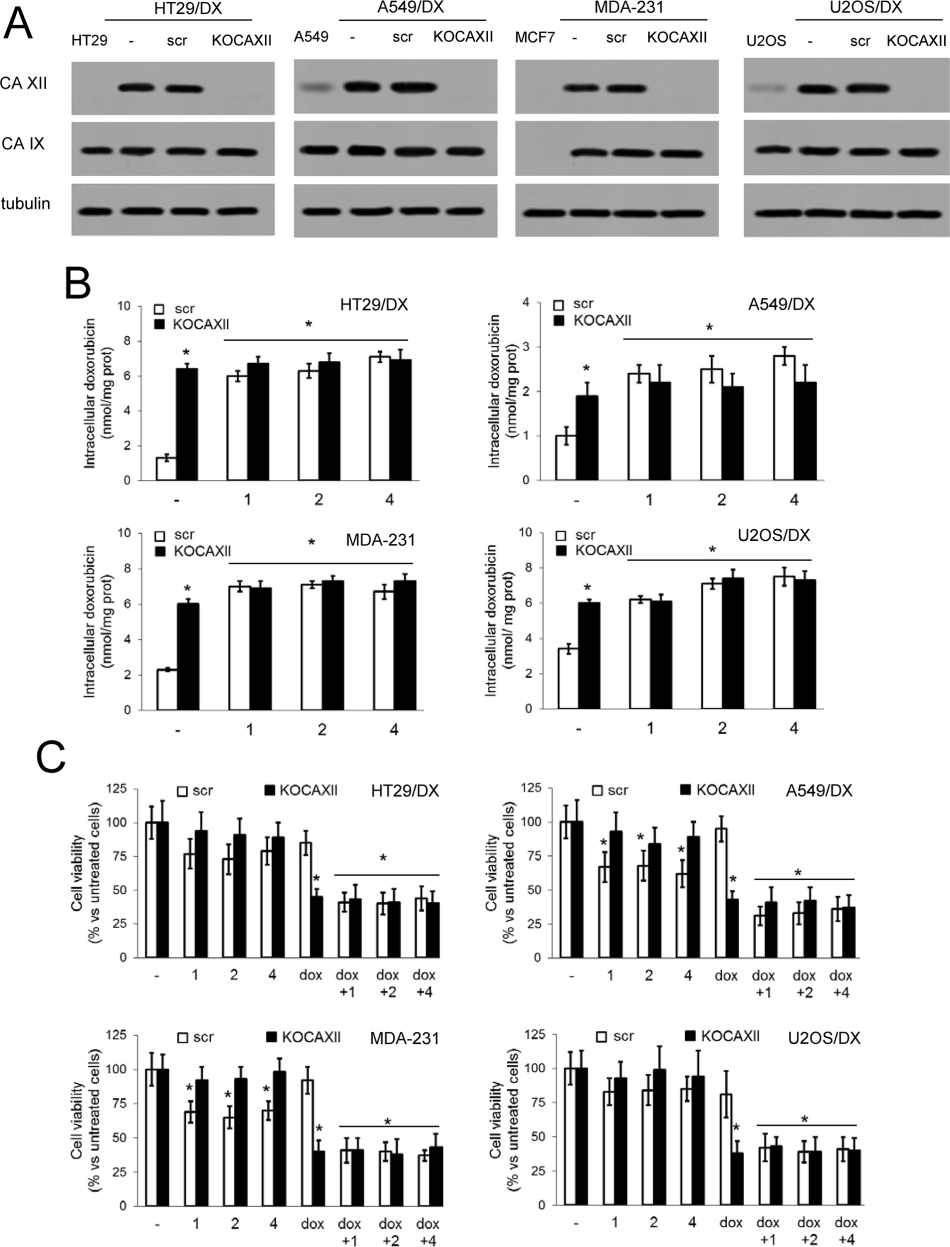
Effects of compound 1, 2 and 4 in *ca12KO* cancer cells (**A**) HT29/DX, A549/DX, MDA-MB-231 and U2OS/DX cells were left untreated (–), treated with a not-targeting scrambled (scr) vector or with a *ca12*-targeting vector (KOCAXII), then immunoblotted for CAXII or CAIX. HT29, A549, MCF, U2OS cells were used as control of cells with low or undetectable levels of CAXII. β-tubulin level was used as control of equal protein loading. The figure is representative of one out of three experiments with similar results. (**B**) Cells treated as in A were incubated for 24 h with 5 μM doxorubicin (–), alone or in the presence of 5 nM compounds 1, 2 or 4, then the intracellular drug content was measured fluorimetrically. Data are presented as means ± SD (*n* = 4). For all cell lines, versus scr cells incubated with doxorubicin alone (–): *p < 0.001. (**C**) Cells were incubated for 72 h in fresh medium (–), or in medium containing 5 μM doxorubicin (dox), 5 nM compounds 1, 2 or 4, alone or in combination, then stained with neutral red dye. The absorbance of viable cells was measured spectrophotometrically. Data are presented as means ± SD (*n* = 4). For all cell lines, versus scr untreated cells (–): *p < 0.01.

**Figure 6:**
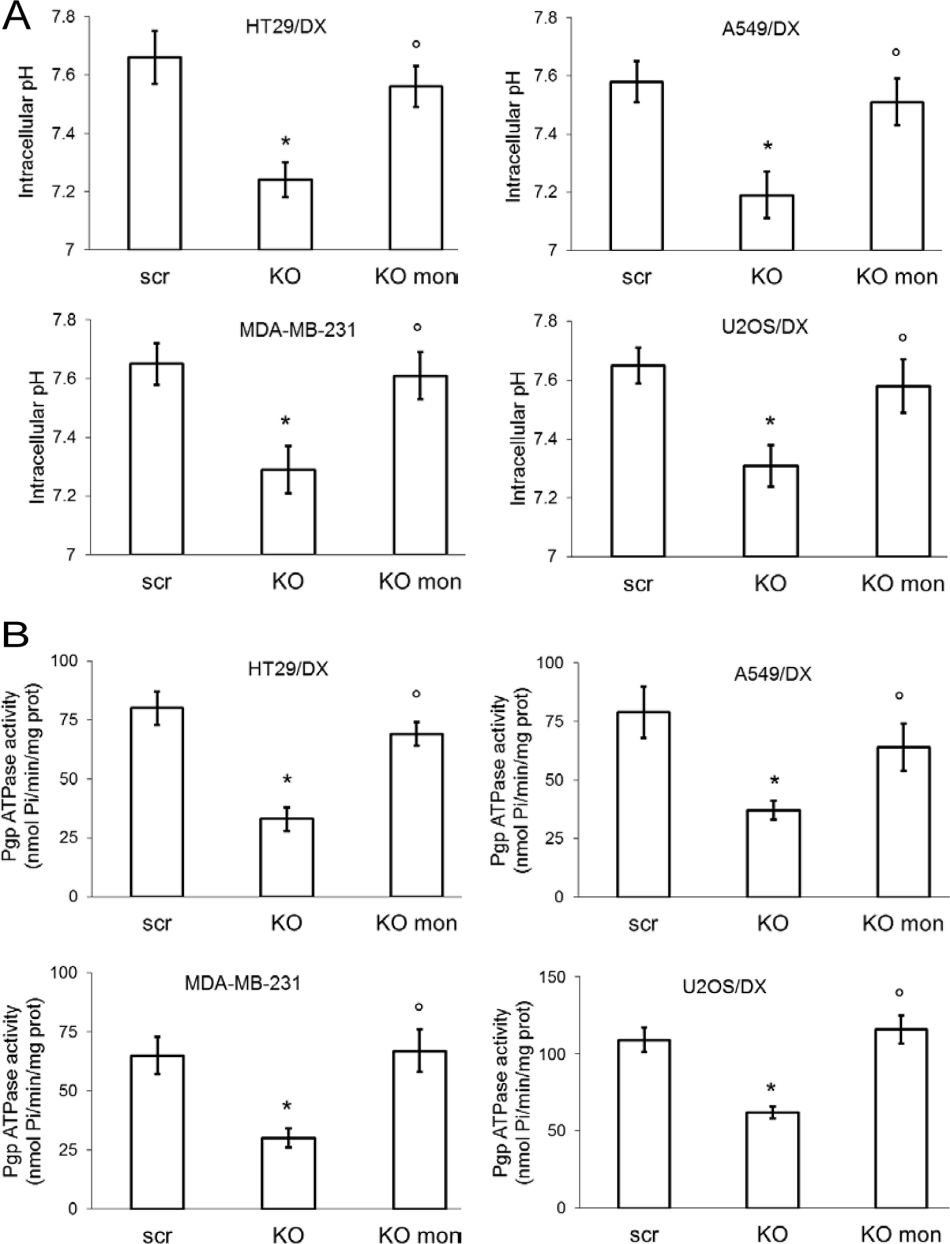
Effects of pH_i_ rescue on Pgp activity of *ca12* KO cells HT29/DX, A549/DX, MDA-MB-231 and U2OS/DX cells were treated with a not-targeting scrambled (scr) vector or with a *ca12*-targeting vector (KO). (**A**) Cells were grown in fresh medium (–) or treated with monensin (10 μM for 3 h; mon). pH_i_ measurement was performed by flow cytometry. Data are presented as means ± SD (*n* = 4). For all cell lines, versus untreated scr cells (–): *p < 0.005; vs KO cells: °*p* < 0.002. (**B**) The Pgp ATPase activity was measured spectrophotometrically on Pgp-rich vesicles extracted from membrane fractions of cells incubated as reported in A. Data are presented as means ± SD (*n* = 3). For all cell lines, versus untreated scr cells (–): * *p <* 0.001; vs KO cells: °*p* < 0.001.

To confirm that the chemosensitizing effect of compounds 1, 2 and 4 was dependent on CAXII activity, we knocked-out *ca12* in a selection of the CAXII-positive and Pgp-positive cell lines: HT29/DX, A549/DX, MDA-MB-231 and U2OS/DX cells (Figure 5A). Compounds 1, 2 and 4 lost the ability to further increase the doxorubicin intracellular accumulation (Figure 5B) in *ca12* knocked-out (KO) cells, to resensitize the cell lines to doxorubicin-induced cytotoxicity (Figure 5C) and to inhibit Pgp activity (Supplementary Figure S8). These results are consistent with the attribution of the effect of compounds 1, 2 and 4 as being dependent on the modulation of CAXII activity. *Ca12* KO cells had lower pH_i_ (Figure 6A) and lower Pgp activity (Figure 6B) than wild-type cells. The addition of the Na^+^/H^+^ ionophore monensin (10 μM) increased the pH_i_ of *ca12* KO cells (Figure 6A) and the Pgp activity (Figure 6B) to values comparable with wild-type cells. Distinct from wild-type cells, doxorubicin did not increase the activity of Pgp in *ca12* KO cells (Supplementary Figure S8), suggesting that the absence of CAXII hampered the substrate-dependent activation of Pgp. In contrast, tariquidar inhibited Pgp activity in both wild-type and *ca12* KO cells (Supplementary Figure S8). These findings suggest that the absence or presence of CAXII does not influence the efficacy of tariquidar.

### CAXII inhibition reduces the growth of doxorubicin-resistant tumors *in vivo*

To test the efficacy of CAXII inhibition in preclinical models of breast resistant tumors, we focused our attention on compound 1, as it had achieved the most striking effect in terms of CAXII inhibition and doxorubicin-chemosensitizing effects *in vitro*.

**Figure 7:**
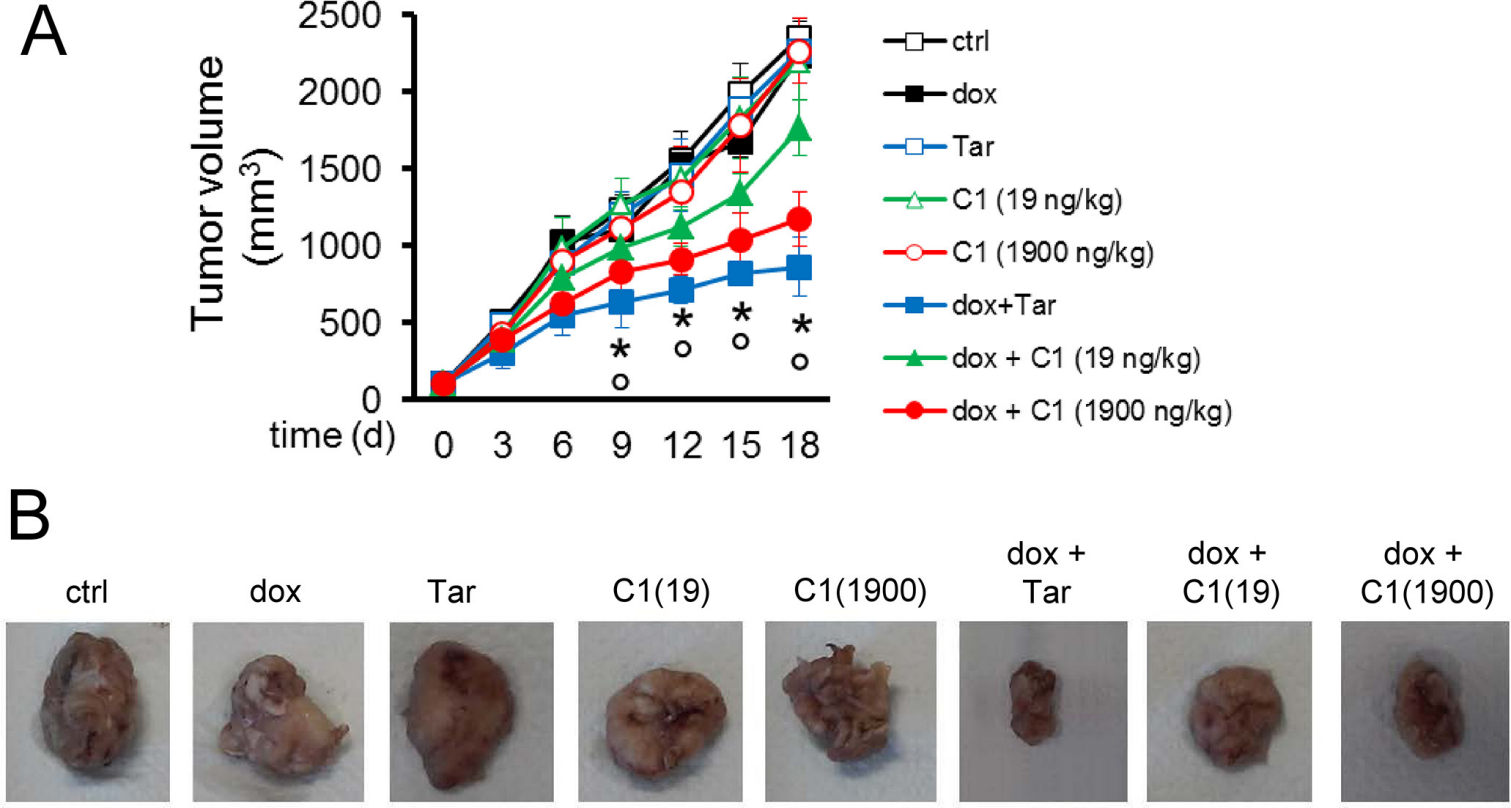
*In vivo* efficacy of compound 1 and doxorubicin against drug-resistant breast tumors (**A**) Six week-old female BALB/c mice were inoculated s.c. with 1 × 10^6^ JC cells. When the tumor reached the volume of 100 mm^3^ (day 7), the mice were randomized into 8 groups (10 animals/group) and treated on days 0, 6 and 12 after the randomization as it follows: 1) control group, treated with 0.1 ml saline solution intravenously (i.v.); 2) doxorubicin group, treated with 5 mg/kg doxorubicin i.v.; 3) tariquidar group, treated with 5 mg/kg tariquidar *per os*; 4) C1 19 group, treated with 19 ng/kg compound 1 (in 0.1 ml saline solution; final concentration: 10 nM) intratumorally; 5) C1 1900 group, treated with 1900 ng/kg compound 1 (in 0.1 ml saline solution; final concentration: 1 μM) intratumorally; 6) doxorubicin + tariquidar group, treated with 5 mg/kg doxorubicin i.v. and 5 mg/kg tariquidar *per os;* 7) doxorubicin + C1 19 group, treated with 5 mg/kg doxorubicin i.v. and 19 ng/kg compound 1 intratumorally; 8) doxorubicin + C1 1900 group, treated with 5 mg/kg doxorubicin i.v. and 1900 ng/kg compound 1 intratumorally. Tumor growth was monitored by caliper measure. Data are presented as means ± SD. Dox +Tar/dox + C1 19/dox + C1 1900 groups versus ctrl group: *p < 0.05; dox + Tar/dox + C1 0.5/dox + C1 50 groups versus dox group: °*p* < 0.005. (**B**) Photographs of representative tumors from each treatment group after mice sacrifice.

We used the highly chemoresistant JC breast tumor model, which is refractory to doxorubicin (Figure 7A–7B). To maximize the drug delivery to the tumor, we injected compound 1 intratumorally, at 10 nM (19 ng/kg) and 1 μM (1900 ng/kg). The lower concentration falls in the range of the CAXII *in vitro* inhibition of compound 1 (*K*_i_ = 1.0 nM); the higher concentration was selected to mitigate compound 1 clearance through lymphatic and blood vessels. Compound 1 has low mouse plasma stability (t_1/2_ = 0.7 h) but good mouse liver microsome stability (t_1/2_ = 134.8 min). At both dosages compound 1 had no effect on tumor growth when used alone, but when co-administered with doxorubicin it significantly increased the anti-proliferative effect of doxorubicin with 21% and 47% reduction in tumor volume at day 18, when used at 19 ng/kg and 1900 ng/kg, respectively (Figure 7A–7B). At 1900 ng/kg compound 1 produced tumor reduction similar to tariquidar (Figure 7A–7B). Worthy of note, at both dosages compound 1 did not induce liver, kidney or heart toxicity, according to the hematochemical parameters of the animals at the time of sacrifice (Supplementary Table S1). As expected, doxorubicin increased creatine phosphokinase (CPK) level, an index of cardiac damage, but compound 1 did not further increase this parameter in doxorubicin-treated animals (Supplementary Table S1).

## DISCUSSION

In this work, we assessed a panel of CAXII inhibitors for their capacity to reverse doxorubicin resistance in MDR cancer cells that overexpress Pgp and resensitize cells to doxorubicin.

Our group has previously designed and synthesized carbohydrate-based CA inhibitors to preferentially inhibit cancer-associated CA isozymes (CAIX and CAXII) over intracellular CAs [12, 20, 21]. The combination of enzyme inhibition and physicochemical properties of the inhibitors was optimized to reduce passive membrane diffusion and favour selective inhibition of extracellular CAs over intracellular CAs. For this study a panel of eight compounds, 1–8, were selected. The cLog *P value* of a compound is indicative of passive membrane permeability, with cLog *P value*s < 0 expected to give compounds with minimal passive cell membrane permeability. Compounds with strong CAXII inhibition, with CAXII selectivity, and with cLog *P value*s < 0 are thus predicted to inhibit extracellular CA enzymes over intracellular CA enzymes and perform best in the cell-based models. The selection of compounds for this study was guided by these parameters.

Compounds 1, 2 and 4–8 all have *K*_i_ < 10 nM for CAXII. These compounds also incorporate a polar monosaccharide or disaccharide moiety such that cLog *P value*s are in the range −3.17 to −4.94. Compound 3 (cLog P + 1.40) is an acyl ester prodrug of compound 2, unlike 2 it is expected that 3 will enter cells by passive membrane diffusion and then be converted to 2 by intracellular esterases. Consequently compounds 2 and 3 provide a ‘drug/prodrug’ pair where the observed activity of the ‘drug’ may be attributed to extracellular (2) and intracellular (3) localization of the inhibitor, respectively.

Compounds 1, 2 and 4 increased doxorubicin accumulation and efficacy in cancer cells that co-express CAXII and Pgp, with the same efficacy of tariquidar, one of the strongest third-generation Pgp inhibitors known [22], used in phase I and phase II clinical trials (https://clinicaltrials.gov). The compounds also increased the intracellular accumulation of vinblastine and paclitaxel, Pgp substrates with alternate Pgp binding sites to doxorubicin [19]. This suggests that the CAXII inhibitors reduce Pgp activity, in a non-Pgp substrate specific manner. Additionally, doxorubicin and vinblastine are weakly basic drugs with increased protonation at acidic pH, while paclitaxel is a non-ionizable drug that remains neutral at acidic pH, hence the resensitizing effect of CAXII inhibitors is not limited to protonatable Pgp substrates. Furthermore, this effect was observed in tumor cells of different histological origins and species, suggesting that the compounds activity is independent of tumor type, species type or Pgp substrate type, requiring only a CAXII positive and Pgp positive phenotype. This phenotype may identify chemoresistant tumors that represent the best candidates for the co-treatment of Pgp substrate cancer chemotherapeutics with CAXII inhibitors. Since CAXII is usually poorly expressed in most healthy cells [5], the use of CAXII inhibitors, may provide a selective tumor-targeting approach when administered with standard chemotherapeutic drugs to patients with drug resistant tumors. Indeed, our compounds were not cytotoxic in epithelial and fibroblasts cells, that had undetectable levels of CAXII. The lack of efficacy of compounds 1, 2 and 4 in *ca12* KO cancer cells further demonstrated that CAXII inhibition is the critical mechanism of action in reversing doxorubicin resistance. Similarly, the lack of efficacy of the compounds in CAXII-negative cells overexpressing Pgp suggests that the increased doxorubicin retention is not due to a direct inhibition of Pgp, but it is a consequence of CAXII inhibition.

CAIX has been correlated with an aggressive tumor phenotype [5, 11] and resistance against doxorubicin in breast cancer tumors [23]. However, in our *in vitro* screening the efficacy of compounds 1, 2 and 4 in increasing doxorubicin accumulation and cytotoxicity was independent of the expression of CAIX. These findings correlate with previous data that demonstrated silencing of CAIX in doxorubicin-resistant cells did not impact on drug-resistance, while silencing of CAXII reversed the drug-resistant phenotype [16].

CAXII is critical for maintaining normal pH_i_ in cancer cells [1, 11]. We hypothesize that the inhibition of CAXII alters the membrane microenvironment where Pgp works, impairing the optimal conditions for its function as an efflux pump. Chemoresistant cells have a slightly alkaline pH_i_ that contributes to the maintenance of their drug-resistant phenotype [24]. Isolated Pgp has consistent basal activity across the pH range 6.2 to 7.6 (at 37°C) [25], which is compatible with the pH_i_ of the doxorubicin-resistant cells analyzed (ranging from 7.45 to 7.64 pH units). We previously reported that the treatment with the classical CAXII inhibitor acetazolamide (AZA), as well as CAXII silencing, lowered the pH_i_ of doxorubicin-resistant colon cancer cells and reduced Pgp activity [16]. Compounds 1, 2 and 4 decreased pH_i_ (1 by 0.24 units, 2 by 0.12 units, and 4 by 0.06 units). Similarly *ca12* KO cells had lower pH_i_ than wild-type cells. This decrease was paralleled by a reduction in Pgp ATPase activity. Of note, when pH_i_ in *ca12* KO cells was increased to the same pH value of wild-type cells with monensin, Pgp activity also increased. These results suggest that the increased pH_i_ maintained by CAXII in resistant cells is critical to maintain optimal Pgp activity. The lower pH_i_ produced by CAXII inhibition reduces Pgp activity, resulting in increased intracellular retention of doxorubicin, vinblastine and paclitaxel. Although we did not measure the pH within the lipid microenvironment where Pgp and CAXII operate in close proximity, we may infer that the inhibition of CAXII reduced the pH in the plasma membrane microenvironment even more than in whole cell, markedly decreasing Pgp activity. Notably, compounds 1, 2 and 4 are all expected to have low cell membrane passive diffusion and thus inhibit CAXII. By contrast, compound 3, a lipophilic ester ‘prodrug’ of 2 and expected to yield compound 2 in the intracellular environment following esterase processing, was devoid of efficacy. These data further suggest that it is inhibition of extracellular facing CAXII (and not intracellular CAs) that may be attributed to the reversal of drug resistance.

Compared with the classical CAXII inhibitor AZA, compounds 1, 2 and 4 offer the advantage of higher selectivity toward CAXII, lower *K*_i_ values and reduced lipophilicity. These attributes render them promising candidates to be tested *in vivo*, using the Pgp-positive and doxorubicin-resistant JC model [26, 27]. We selected compound 1 for an *in vivo* study as this compound had the maximal chemosensitizing effect *in vitro*.

To avoid compound related metabolism or clearance and maximise tumor delivery, we administered 1 intratumorally. Either a dosage of 1 around the CAXII *K*_i_ or a 100-fold higher dosage, to counteract compound clearance through lymphatic and blood vessels, significantly reduced tumor growth when co-administered with doxorubicin. Since the compound alone (i.e. without co-administration of doxorubicin) did not reduce tumor growth, such effect was likely due to the increased retention of doxorubicin within cancer cells. These data suggest that compound 1 effectively reverses the resistance to doxorubicin *in vivo* to resensitize these resistant cells to doxorubicin treatment. The sensitization efficacy was comparable to the third-generation Pgp inhibitor tariquidar. Moreover, compound 1, either alone or with doxorubicin, did not elicit liver or kidney toxicity, or increase the cardiac damage induced by doxorubicin (an anthracycline), according to the hematochemical parameters of the animals. We propose that the indirect mechanism of action of CAXII inhibitors to reduce Pgp activity voids the usual *in vivo* toxicity associated with direct Pgp inhibition. Additionally, as CA inhibitors have been a mainstay of human clinical intervention for several decades (more than 25 clinically approved CA inhibitors) this drug class has a recorded safety profile [28]. Moreover, mammalian CAs belong to the α-CA family, with murine CAXII expressed in the large intestine and kidney similarly to human CAXII [29, 30]. Given the significant chemosensitizing effect of compound 1, we speculate that the dosage of doxorubicin- if co-administered with compound 1 (or other selective small molecule CAXII inhibitors) -may even be reduced while retaining its antitumor efficacy yet limiting doxorubicin associated cardiotoxicity.

In summary, our work suggests that CAXII inhibitors are potent chemosensitizing agents in tumors overexpressing both CAXII and Pgp. Given the prevalent expression of CAXII in tumor tissues and the high selectivity of our inhibitors towards this CA isoform, our compounds appear as promising tumor-selective agents. These results may be a turning point toward new treatments based on a combination of a CAXII selective inhibitor with chemotherapeutic drugs, where the chemotherapeutic drugs alone are ineffective in treating Pgp-positive (i.e. MDR) tumors. This approach may be particularly beneficial for patients with tumors that co-express CAXII and Pgp, a typical feature of aggressive and chemoresistant tumors. Furthermore, neo-adjuvant protocols based on anthracyclines are currently used in the treatment of breast cancer to improve patient outcomes, with the purpose to reduce tumor mass and allow a more conservative surgery [31]. In a translational perspective our study may provide the rational basis for a new neo-adjuvant combinatorial therapy for breast tumors to enhance the effectiveness of cytotoxic chemotherapeutic drugs, i.e. the loco-regional administration of a CAXII inhibitor as a chemosensitizer agent followed by systemic administration of doxorubicin. To the best of our knowledge, such an approach has not been reported. The expression of CAIX, the more well studied cancer-associated CA isozyme, did not influence the effects of the compounds on the intracellular doxorubicin accumulation in all cell lines tested. We recommend that future studies involving CAs in cancer concomitantly examine CAIX and CAXII expression, rather than only CAIX, to better inform the interpretation of experimental findings.

## MATERIALS AND METHODS

### Materials

The plasticware for cell cultures was obtained from Falcon (Becton Dickinson, Franklin Lakes, NJ). The electrophoresis reagents were obtained from Bio-Rad Laboratories (Hercules, CA). The protein content of cell lysates was assessed with the BCA kit from Sigma Chemicals Co. (St. Louis, MO). Tariquidar dihydrochloride was from Tocris Bioscience (Bristol, UK). Unless specified otherwise, all reagents were purchased from Sigma Chemicals Co.

### CAXII inhibitors

The synthesis and structural characterization of carbohydrate-based sulfamates (1–4) [32, 33] and sulfonamides (5–8) [12] has been fully described previously by us. The chemical structure of the compounds is reported in Figure 1.

### CA inhibition

The inhibition of CA I, II, IX and XII by test inhibitors (1–8) has been previously reported by us [12, 32, 33]. Inhibition data and isozyme selectivity values are reported in Table 1. Briefly, a stopped-flow instrument was used to monitor the CA-catalyzed CO_2_ hydration reaction for a period of 10–100 s with phenol red as a pH indicator. Saturated CO_2_ solutions in water at 20°C were used as substrate, while the recombinant CA protein was prepared in Hepes buffer (10 mM, pH 7.5) with added NaClO_4_ (0.1 M) to maintain a constant the ionic strength. The inhibitor and enzyme solutions were preincubated for 15 min at room temperature prior to assay in order to allow for the formation of the enzyme-inhibitor complex. The inhibition constants were obtained by nonlinear least-squares methods using PRISM 3. The curve fitting algorithm allowed us to obtain the IC_50_ values, from which *K*_i_ values were calculated by using the Cheng-Prusoff equation.

### Cell lines

Human colon cancer HT29 cells, lung cancer A549 cells, breast cancer MCF-7, SKBR3, T74D and MDA-MB-231 cells, osteosarcoma U2OS and SaOS cells, murine chemoresistant JC cells, not-transformed human colon epithelial CCD-Co-18 cells, lung epithelial BEAS-2B cells, breast epithelial MCF10A cells were purchased from ATCC (Manassas, VA). Murine chemoresistant TUBO cells were a kind gift of Prof. Federica Cavallo, Department of Molecular Biotechnology and Health Sciences, University of Torino, Italy. Not-transformed human fibroblasts were a kind gift of Prof. Franco Novelli, Department of Molecular Biotechnology and Health Sciences, University of Torino, Italy. Human HT29/DX and A549/DX were generated by stepwise selection in medium with increasing concentration of doxorubicin, as reported by us [34], and maintained in culture medium with a final concentration of 200 nM and 100 nM doxorubicin, respectively. U2OS/DX and SaOS/DX cells were generated with a similar procedure [35] and maintained in culture medium with a final concentration of 1 μM doxorubicin. The expression levels of CAXII, CAIX and Pgp were measured by immunoblotting (see below): the results are reported in the Supplementary Figure S1. All cell lines were authenticated by microsatellite analysis, using the PowerPlex kit (Promega Corporation, Madison, WI; last authentication: June 2016). Cells were maintained in media supplemented with 10% v/v fetal bovine serum, 1% v/v penicillin-streptomycin, 1% v/v L-glutamine.

### Immunoblotting

For whole cell lysates, the cells were rinsed with ice-cold lysis buffer (50 mM, Tris, 10 mM EDTA, 1% v/v Triton-X100), supplemented with the protease inhibitor cocktail set III (80 μM aprotinin, 5 mM bestatin, 1.5 mM leupeptin, 1 mM pepstatin; Calbiochem, San Diego, CA), 2 mM phenylmethylsulfonyl fluoride and 1mM Na_3_VO_4_, then sonicated and centrifuged at 13,000× g for 10 min at 4°C. 20 μg protein extracts were subjected to SDS-PAGE and probed with the following antibodies: anti-CAXII (Abcam, Cambridge, UK), anti-CAIX (Novus Biologicals, Littleton, CO), anti-Pgp (C219, Calbiochem), anti-β-tubulin (Santa Cruz Biotechnology Inc., Santa Cruz, CA), followed by a peroxidase-conjugated secondary antibody (Bio-Rad Laboratories). The membranes were washed with Tris-buffered saline-Tween 0.1% v/v solution, and the proteins were detected by enhanced chemiluminescence (Bio-Rad Laboratories). Plasma membrane-associated proteins were evaluated in biotinylation assays, using the Cell Surface Protein isolation kit (Thermo Fisher Scientific Inc., Waltham, MA), as previously reported [36]. An anti-pancadherin antibody (Santa Cruz Biotechnology Inc.) was used to confirm equal protein loading. In co-immunoprecipitation experiments, 100 μg of plasma membrane-associated proteins were immunoprecipitated with the anti-Pgp antibody, using the PureProteome protein A and protein G Magnetic Beads (Millipore, Billerica, MA). The immunoprecipitated proteins were separated by SDS-PAGE and probed with anti-CAXII or anti-Pgp antibodies, followed by a peroxidase-conjugated secondary antibody.

### Pgp overexpression

To generate the Pgp-positive MCF7 and SKBR3 cells, the pHa vector containing the complete *mdr1* cDNA was purchased from Addgene (Cambridge, MA) and subcloned into pCDNA3 vector (Invitrogen Life Technologies, Milan, Italy) as described [37].

### Chemotherapeutic drug accumulation

Doxorubicin content was measured fluorimetrically as detailed previously by us [34]. Vinblastine and paclitaxel accumulation were measured by labelling cells with 1 μCi [^3^H]-vinblastine sulphate (PerkinElmer, Waltham, MA) and [^3^H]-paclitaxel (Moravek Inc., Brea, CA). Cells were washed twice with PBS, detached with trypsin and sonicated. The intracellular drug content was measured by liquid scintillation. The results were expressed as nmol drug/mg cell proteins, according to titration curves previously set.

### Cell viability

Cell viability was evaluated by measuring the percentage of cells stained with neutral red dye, as reported previously [38]. The viability of untreated cells was considered 100%; the results were expressed as percentage of viable cells in each experimental condition versus untreated cells. To determine the IC_50_ of doxorubicin in CAXII inhibitor treated cells 5 × 10^5^ cells, with concentrations of doxorubicin (ranging from 1 nM to 1 mM), were incubated in the absence or presence of 5 nM of each CAXII inhibitor for 72 h. The inhibitory concentration 50 (IC_50_) is defined as the concentration of doxorubicin that kills 50% of cells. The resistance factor (Rf) was calculated as the ratio between mean IC_50_ in cells treated with CAXII inhibitors and mean IC_50_ in cells treated with tarquidar.

### Pgp ATPase activity

The assay was performed on Pgp-enriched membrane vesicles as detailed in [39]. Verapamil (10 μM) was added to the reaction mix to achieve a maximal activation of the Pgp ATPase activity. Results were expressed as nmol hydrolyzed phosphate (Pi)/min/mg proteins, according to the titration curve previously prepared.

### pH_i_ measurement

pH_i_ was measured by incubating whole cells with 5 μM 2′,7′-bis-(2-carboxyethyl)-5-(and-6)-carboxyfluorescein acetoxymethyl ester for 15 min at 37°C and reading the intracellular fluorescence by a FACSCalibur flow cytometer (Becton Dickinson). The intracellular fluorescence was converted into pH units according to a titration curve, as described previously [24].

### Ca12 knockout

5 × 10^5^ cells were transduced with 1 μg RNA vector (CRISPR pCas guide vector) ca12 or 1 μg not-targeting vector, mixed with 1 μg donor DNA vector (Origene, Rockville, MD), following the manufacturer's instructions. Stable KO cells were selected in complete medium containing 1 μg/ml puromycin for three weeks. Knockout efficacy was evaluated by immunoblotting, as reported above. Cell viability was evaluated by neutral red staining: *ca12* KO cells had the same viability than parental cells (data not shown).

### *In vivo* tumor growth

To evaluate the anti-tumor efficacy of CAXII inhibitors, we injected subcutaneously 1 × 10^6^ JC cells, a mammary cancer cell line known for its high expression of Pgp [26] and its strong refractoriness to doxorubicin [27], mixed with 100 μL Matrigel, in syngeneic 6 week-old female BALB/c mice (weight: 20 g ± 1.3; Charles River Laboratories Italia, Calco). Animals were housed (5 per cage) under 12 h light/dark cycles, with food and drinking provided *ad libitum*. Tumor growth was measured daily by caliper and calculated according to the equation (L × W^2^)/2, where L = tumor length, W = tumor width. When the tumor reached a volume of 100 mm^3^ (day 7 after injection), the mice were randomized into 8 groups (10 animals/group) and treated on days 0, 6 and 12 after the randomization as it follows: 1) control group, treated with 0.1 ml saline solution intravenously (i.v.); 2) doxorubicin group, treated with 5 mg/kg doxorubicin i.v.; 3) tariquidar group, treated with 5 mg/kg tariquidar *per os*; 4) C1 19 group, treated with 19 ng/kg compound 1 (in 0.1 ml saline solution; final concentration: 10 nM) intratumorally; 5) C1 1900 group, treated with 1900 ng/kg compound 1 (in 0.1 ml saline solution; final concentration: 1 μM) intratumorally; 6) doxorubicin + tariquidar group, treated with 5 mg/kg doxorubicin i.v. and 5 mg/kg tariquidar *per os*; 7) doxorubicin + C1 19 group, treated with 5 mg/kg doxorubicin i.v. and 19 ng/kg compound 1 intratumorally; 8) doxorubicin + C1 1900 group, treated with 5 mg/kg doxorubicin i.v. and 1900 ng/kg compound 1 intratumorally. Tumor volumes were monitored daily by caliper and animals were euthanized by injecting zolazepam (0.2 ml/kg) and xylazine (16 mg/kg) intramuscle (i.m.) at day 18. Tumors were excised and photographed immediately after mice sacrifice. The hematochemical parameters lactate dehydrogenase (LDH), aspartate aminotransferase (AST), alanine aminotransferase (ALT), alkaline phosphatase (AP), creatinine, CPK were measured on 0.5 ml of blood collected immediately after mice sacrifice, using the respective kits from Beckman Coulter Inc. (Miami, FL). Animal care and experimental procedures were approved by the Bio-Ethical Committee of the Italian Ministry of Health (#122/2015-PR).

### Pharmacokinetics

Plasma stability (mouse) and liver microsome metabolic stability (mouse) were measured by the Biopharmacy Laboratory, Shanghai Institute of Materia Medica, Chinese Academy of Sciences.

### Statistical analysis

All data in the text and figures are provided as means ± SD. The results were analyzed by a one-way analysis of variance (ANOVA) and Tukey's test, using Statistical Package for Social Science (SPSS) software (IBM SPSS Statistics v.19). *p* < 0.05 was considered significant.

## SUPPLEMENTARY FIGURES AND TABLE


