# Supplementary material for: P-glycoprotein-mediated chemoresistance is reversed by carbonic anhydrase XII inhibitors
Source: Oncotarget. 2016 Nov 3;7(52):85861–75. doi: 10.18632/oncotarget.13040 (PMC5349880; doi:10.18632/oncotarget.13040)
Supplement: Supplementary file 1 [file oncotarget-07-85861-s001.pdf]

P-glycoprotein-mediated chemoresistance is reversed by carbonic anhydrase XII inhibitors

Supplementary Materials

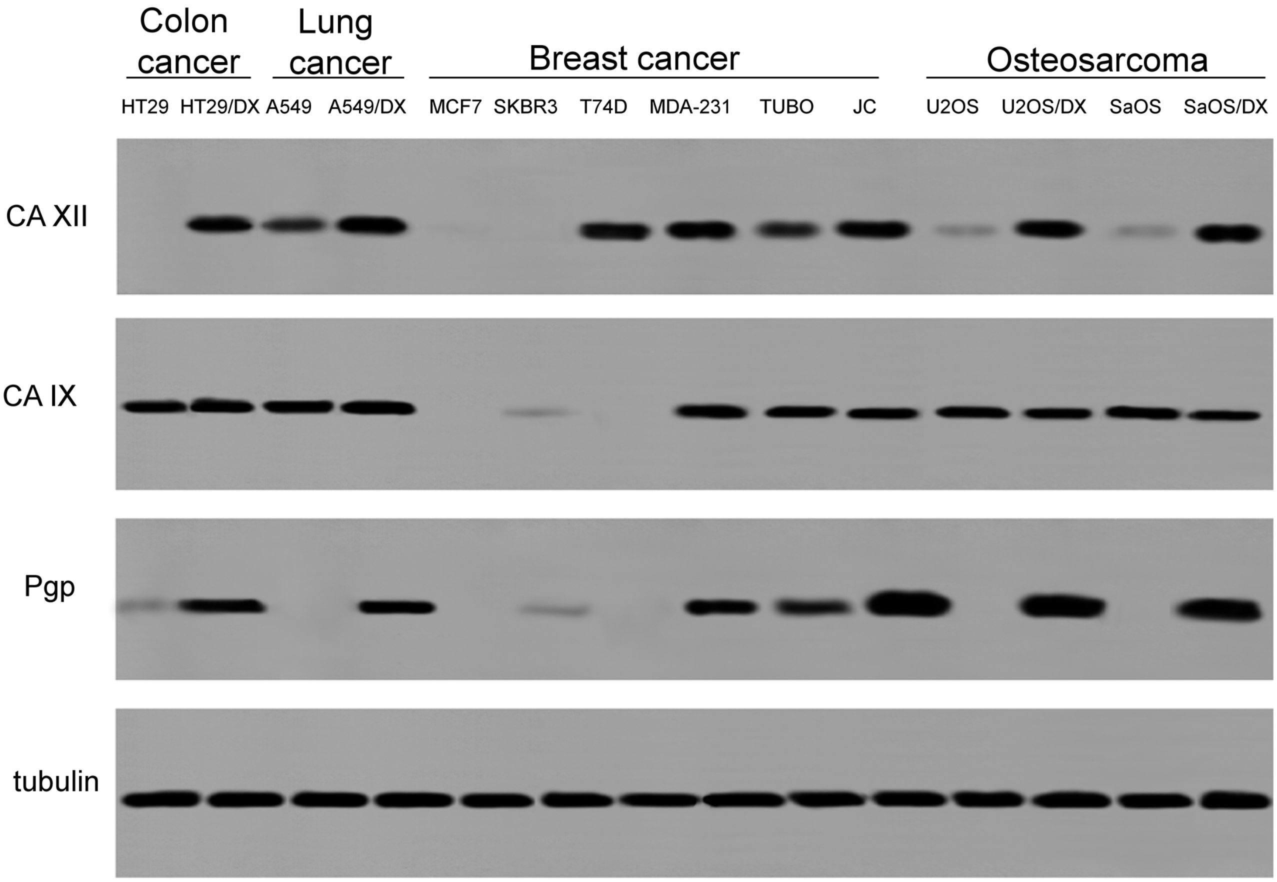

Supplementary Figure S1: Expression of CAXII, CAIX and Pgp in different cancer cells. Human doxorubicin-sensitive colon cancer HT29 cells and their resistant counterpart HT29/DX cells, human doxorubicin-sensitive lung cancer A549 cells and their resistant counterpart A549/DX cells, human doxorubicin-sensitive and resistant breast cancer MCF7, SKBR3, T74D and MDA-MB-231 cells, murine doxorubicin-resistant TUBO and JC cells, human doxorubicin-sensitive osteosarcoma U2OS cells and their resistant counterpart U2OS/DX cells, human doxorubicin-sensitive osteosarcoma SaOS and their resistant counterpart SaOS/DX were lysed and immunoblotted for CAXII, CAIX and Pgp. β-tubulin level was used as control of equal protein loading. The figure is representative of one out of three experiments with similar results.

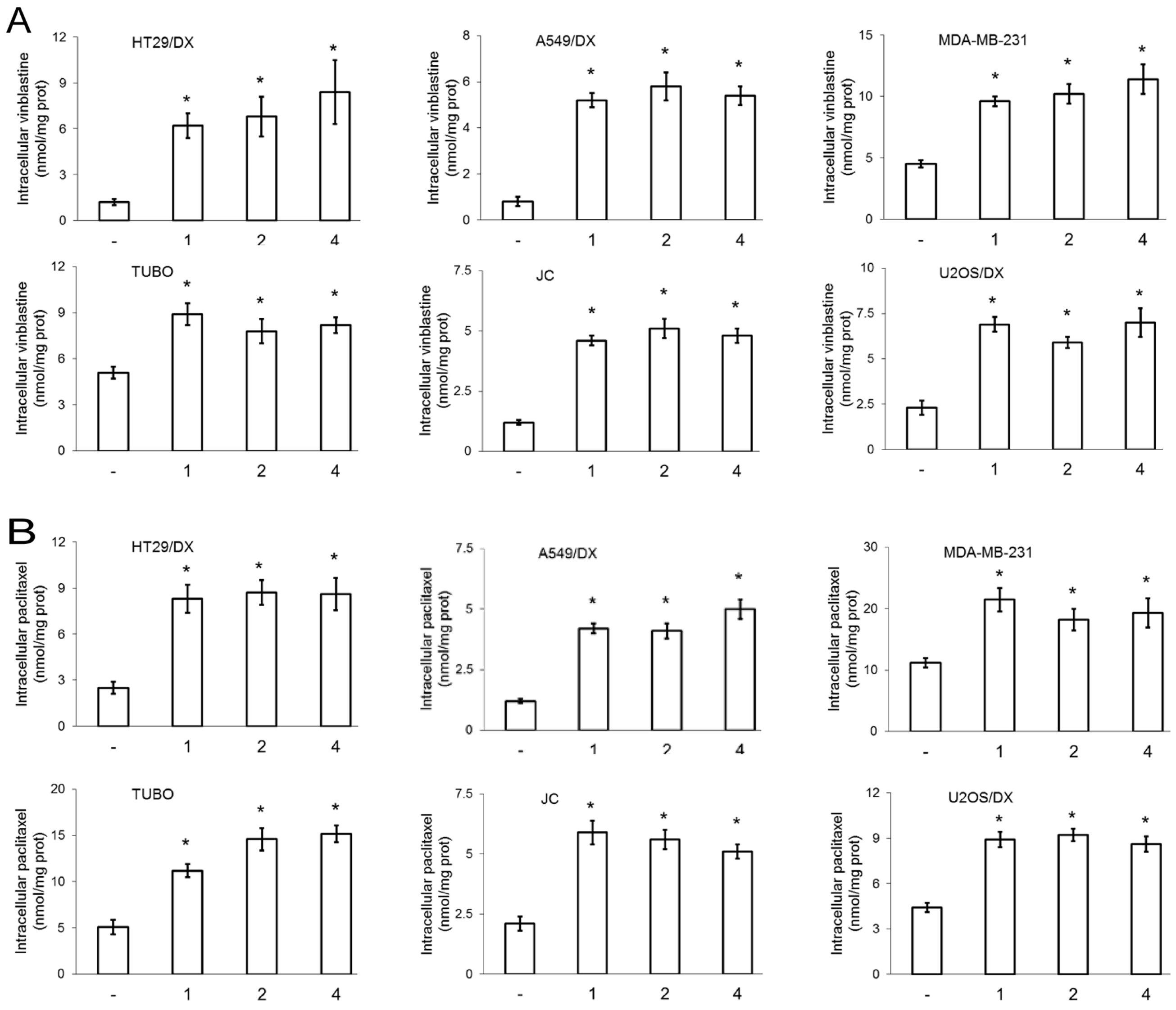

Supplementary Figure S2: Vinblastine and paclitaxel accumulation in CAXII- and Pgp-positive cells. CAXII-positive and Pgp-positive HT29/DX cells, A549/DX cells, MDA-MB-231 cells, TUBO cells, JC cells, U2OS/DX cells were incubated with 1 µCi [^{3}H]-vinblastine (panel **A**) or [^{3}H]-paclitaxel (panel **B**) for 24 h, in fresh medium (–) or in medium containing 5 nM of compounds 1, 2 and 4. The intracellular drug content was measured by liquid scintillation. Data are presented as means ± SD (n = 4). Versus vinblastine/ paclitaxel alone (–): *p < 0.001.

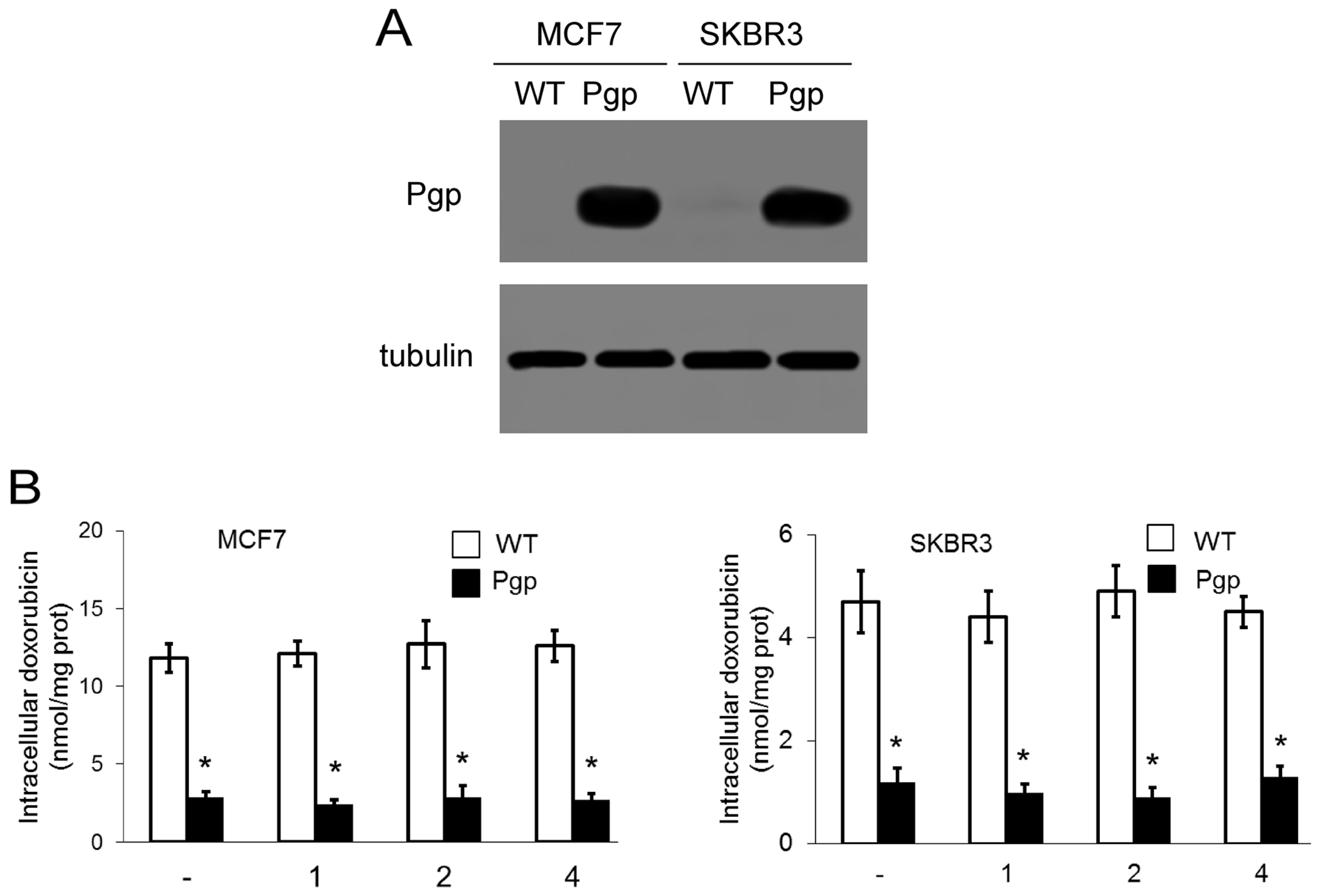

Supplementary Figure S3: Effects of inhibitors on CAXII-negative cells transfected with exogenous Pgp. (**A**) Wild-type (WT) or Pgp-overexpressing (Pgp) MCF7 and SKBR3 cells were lysed and immunoblotted for Pgp. β-tubulin level was used as control of equal protein loading. The figure is representative of one out of three experiments with similar results. (**B**) Cells were grown for 24 h in the presence of 5 μM doxorubicin, alone (–) or in the presence of 5 nM of compounds 1, 2 and 4. The intracellular drug content was measured fluorimetrically. Data are presented as means ± SD ($n = 4$). Versus WT cells: $^{*}p < 0.001$.

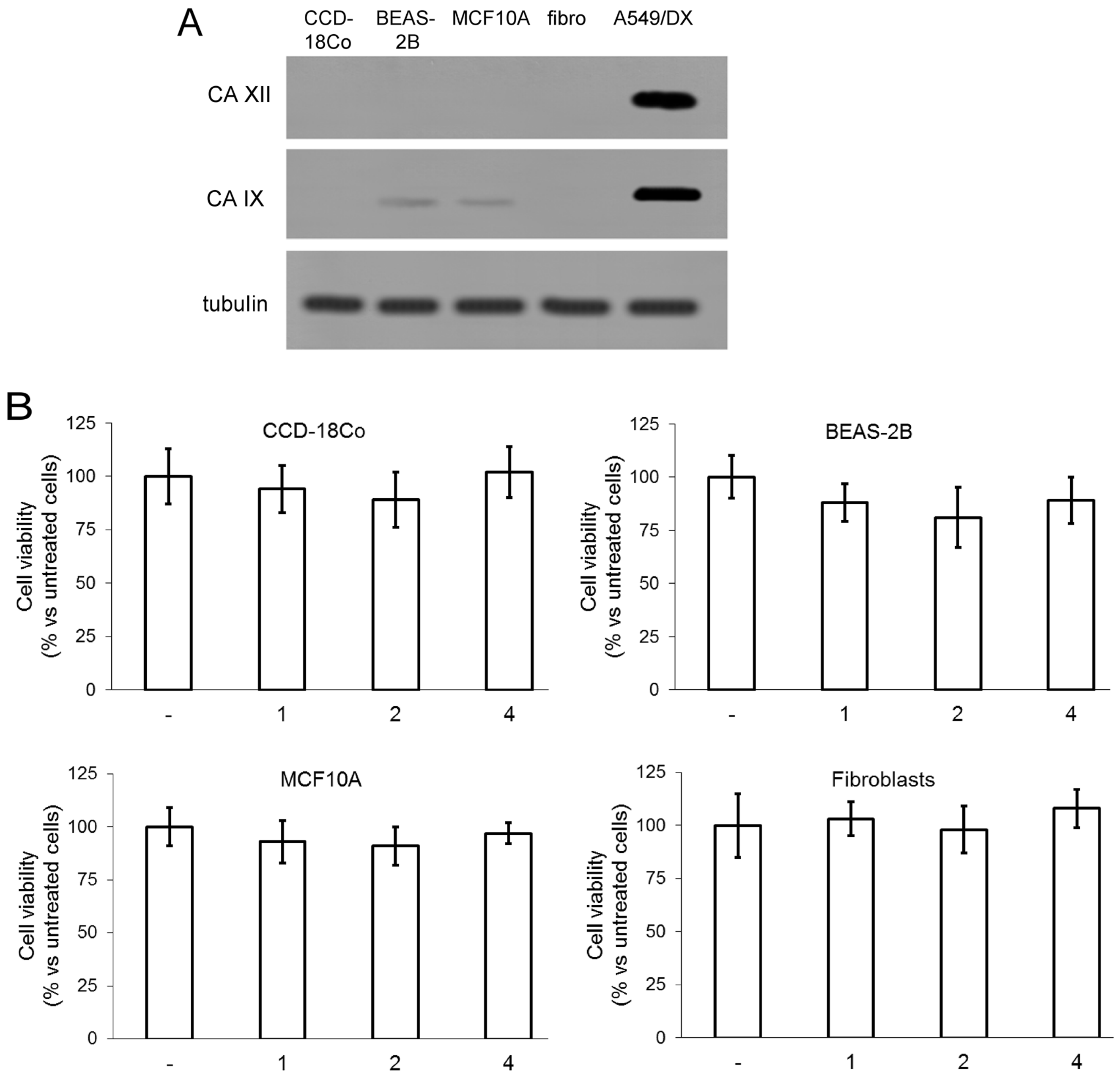

Supplementary Figure S4: Effects of compounds 1, 2 and 4 on not-transformed cells. (**A**) Not-transformed human epithelial colon CCD-Co-18 cells, epithelial lung BEAS-2B cells, epithelial breast MCF10A cells, fibroblasts (fibro) were lysed and immunoblotted for CAXII and CAIX. A549/DX cells were used as control of CAXII- and CAIX-positive cells. β-tubulin level was used as control of equal protein loading. The figure is representative of one out of three experiments with similar results. (**B**) Cells were grown for 72 h in fresh medium (–), or in medium containing 5 nM compounds 1, 2 and 4, then stained with neutral red dye. The absorbance of viable cells was measured spectrophotometrically. Data are presented as means ± SD ($n = 4$).

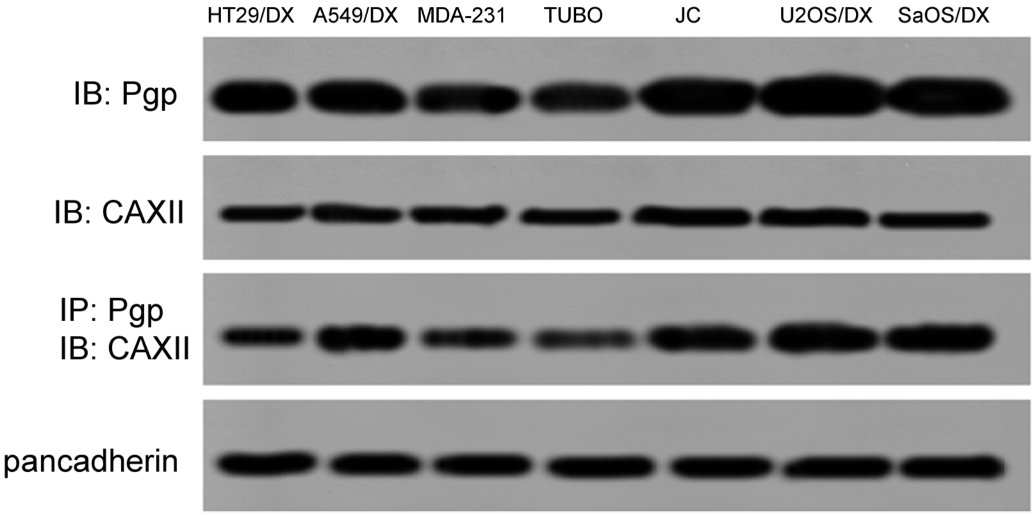

Supplementary Figure S5: Co-immunoprecipitation of Pgp and CAXII in Pgp-enriched vesicles. Pgp-enriched vesicles used for the ATPase assays were checked for the expression of Pgp and CAXII by immunoblotting (IB). When indicated, vesicle extracts were immunoprecipitated (IP) with anti-Pgp antibody, then immunoblottedwith anti-CAXII antibody. Pancadherin level was used as control of equal protein loading.

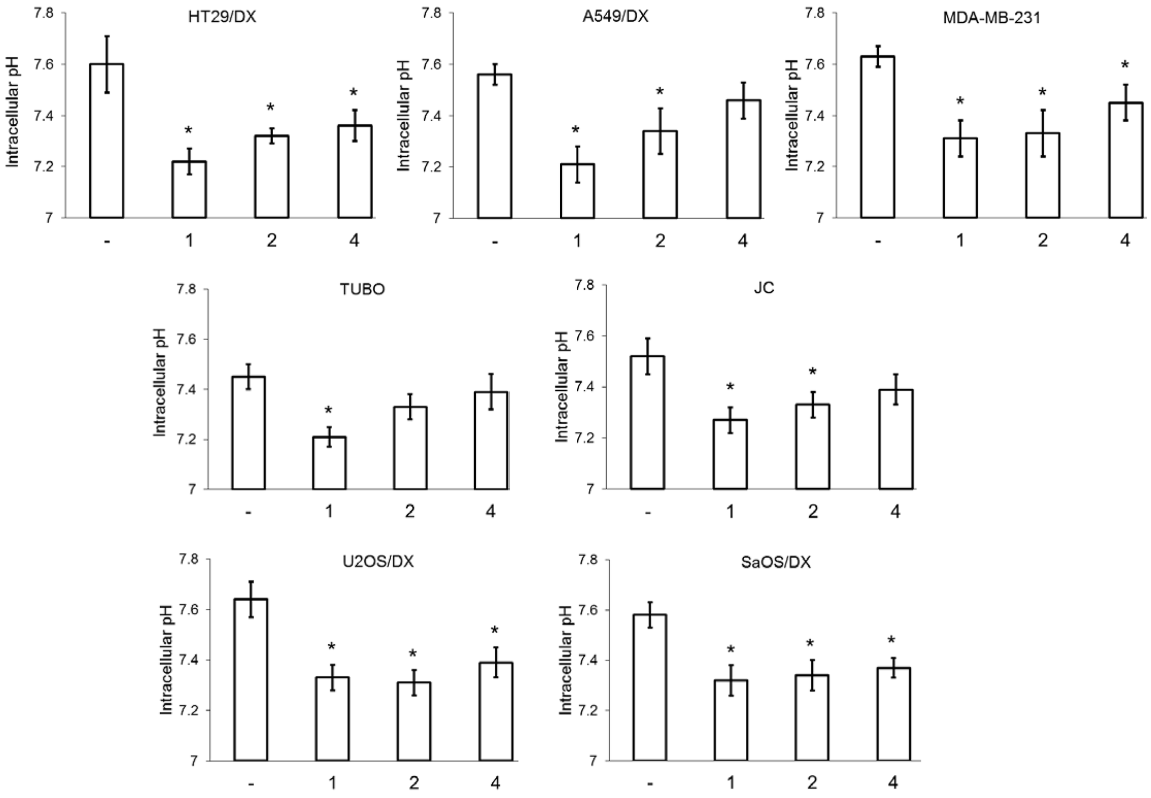

Supplementary Figure S6: Effects of compounds 1, 2 and 4 on intracellular pH. pHi measurement was performed by flow cytometry in HT29/DX, A549/DX, MDA-MB-231, TUBO, JC, U2OS/DX and SaOS/DX cells, untreated (–) or treated for 3 h with 5 nM compounds 1, 2 and 4. Data are presented as means ± SD ($n = 4$). For all cell lines, versus untreated cells (–): $*p < 0.01$.

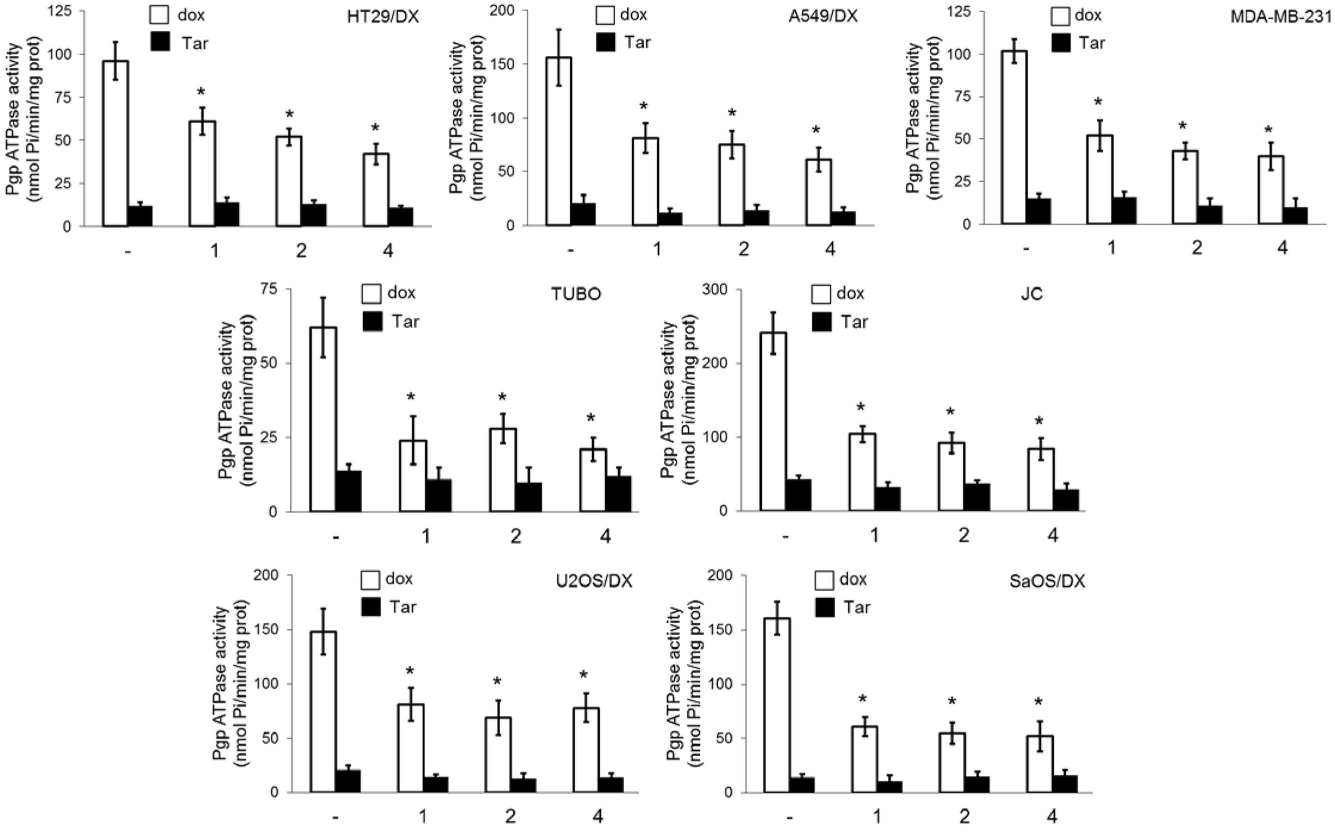

Supplementary Figure S7: ATPase activity in the presence of Pgp substrate and inhibitor. Cells were grown for 24 h in fresh medium (–) or in medium containing 5 nM of compounds 1, 2 and 4. The Pgp ATPase activity was measured spectrophotometrically using Pgp-rich vesicles extracted from membrane fractions. Doxorubicin (5 µM, Dox) and tariquidar (25 nM, Tar) were added during the assay, to measure the ATPase activity in the presence of a Pgp substrate or inhibitor, respectively. Data are presented as means ± SD (*n* = 3). For all cell lines, versus untreated cells (–): $^{*}p < 0.001$.

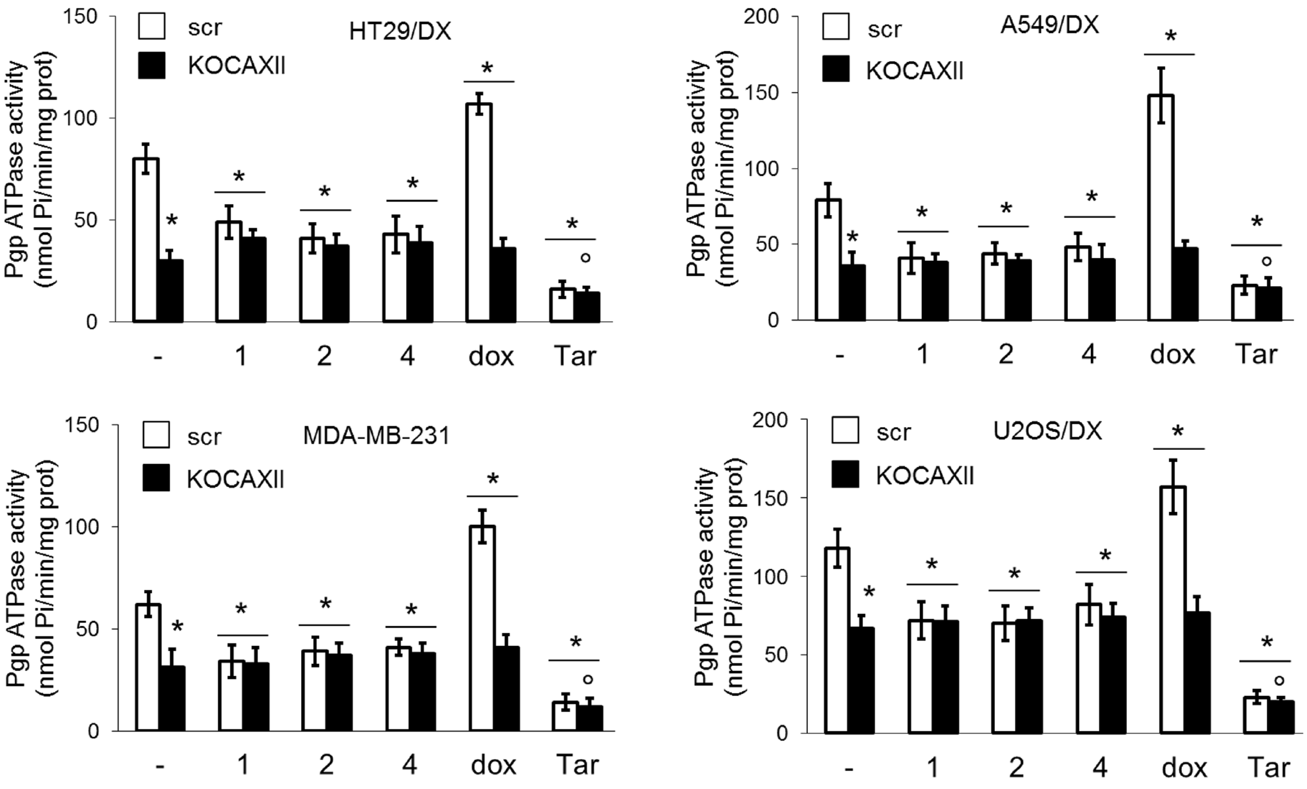

Supplementary Figure S8: Effects of *ca12* knockout on Pgp activity. HT29/DX, A549/DX, MDA-MB-231 and U2OS/DX cells were treated with a not-targeting scrambled (scr) vector or with a *ca12*-targeting vector (KOCAXII). Cells were grown for 24 h in fresh medium (–) or in medium containing 5 nM compounds 1, 2 and 4. When indicated, doxorubicin (5 µM, Dox) and tariquidar (25 nM, Tar) were added during the assay, to measure the ATPase activity in the presence of a Pgp substrate or inhibitor, respectively. The Pgp ATPase activity was measured spectrophotometrically on Pgp-rich vesicles extracted from membrane fractions. Data are presented as means ± SD (n = 3). For all cell lines, versus untreated scr cells (–): * $p < 0.02$; versus untreated KO cells: °$p < 0.05$.

Supplementary Table S1: Hematochemical parameters of animals

	ctrl	dox	C1 (19 ng/kg)	C1 (1900 ng/kg)	dox + C1 (19 ng/kg)	dox + C1 (1900 ng/kg)
LDH (U/l)	6034 ± 1118	6272 ± 812	6723 ± 1023	6729 ± 835	6091 ± 836	6241 ± 936
AST (U/l)	201 ± 32	288 ± 72	239 ± 72	261 ± 71	221 ± 71	182 ± 44
ALT (U/l)	43 ± 13	37 ± 16	38 ± 12	45 ± 7	44 ±11	33 ± 15
AP (U/l)	72 ± 19	824 ± 23	91 ± 13	83 ± 11	79 ± 11	83 ± 19
Creatinine (mg/l)	0.026 ± 0.005	0.029 ± 0.017	0.021 ± 0.011	0.033 ± 0.008	0.029 ± 0.011	0.028 ± 0.008
CPK (U/l)	267 ± 102	673 ± 172*	271 ± 84	312 ± 81	701 ± 129*	691 ± 103*

Animals (n = 10/group) were treated as reported under Materials and methods. Blood was collected immediately after euthanasia and analyzed for lactate dehydrogenase (LDH), aspartate aminotransferase (AST), alanine aminotransferase (ALT), alkaline phosphatase (AP), creatinine, creatine phosphokinase (CPK). ctrl: mice treated with saline solution; dox: mice treated with doxorubicin; C1: mice treated with compound 1. Versus ctrl group: *$p < 0.005$.
